# Supplementary material for: Distinct Effects of High-Fat and High-Phosphate Diet on Glucose Metabolism and the Response to Voluntary Exercise in Male Mice
Source: Nutrients. 2022 Mar 12;14(6):1201. doi: 10.3390/nu14061201 (PMC8951123; doi:10.3390/nu14061201)
Supplement: Supplementary file 1 [file nutrients-14-01201-s001.zip › nutrients-1619725-supplementary.pdf]

Supplementary Table S1. Primer sequences

Gene Name	Forward Primer	Reverse Primer
Prdm16	ATCCACAGCACGGGTGAAGCCAT	ACATCTGCCCACAGTCCTTGCA
Tfam	GAGGCAAAGGATGATTCGGCTC	CGAATCCTATCATCTTTAGCAAGC
Pgc1a	GAATCAAGCCACTACAGACACCG	CATCCCTCTTGAGCCTTTCGTG
Cs	GACTACATCTGGAACACACTCAATTCA	CGAGGGTCAGTCTTCCTCAGTAC
Nrf1	CAACAGGGAAGAAACGGAAA	GCACCACATTCTCCAAAGGT
Nrf2	AGGTTGCCCACATTCCCAAACAAG	TTGCTCCATGTCCTGCTCTATGCT
Fatp4	GTATAGAAGGCGGTGTTGTC	CCAAACCTCAATACATCTAAC
Mcad	GATGCATCACCCTCGTGTAAC	AAGCCCTTTTCCCCTGAA
Glut4	ATCATCCGGAACCTGGAGG	CGGTCAGGCGCTTTAGACTC
Hk2	AGAGAACAAGGGCGAGGAG	GGAAGCGGACATCACAATC
Aldoa	GCGACCACCATGTCTATCTG	GAAAGTGACCCCAGTGACAG
Cd36	TGGAGCTGTTATTGGTGCAG	TGGGTTTTGCACATCAAAGA
